# Creating hot spots within air for better sensitivity through design of oblique-wire-bundle metamaterial perfect absorbers

**DOI:** 10.1038/s41598-022-07338-6

**Published:** 2022-03-03

**Authors:** Xin-Xian Wu, Cheng-Yu Lu, Tsung-Yu Huang

**Affiliations:** Department of Materials Engineering, Ming Chi University of Technology, New Taipei City, 243303 Taiwan

**Keywords:** Metamaterials, Optical sensors, Nanophotonics and plasmonics

## Abstract

Better sensitivity of a biosensor could boost up the detection limit of analytes, thus a must in the fields of bio-sensing and bio-detection. To further enhance the sensitivity of a biosensor, in this work, we design an oblique-flat-sheet metamaterial perfect absorber (MPA) to concentrate the hot spots within air between the oblique flat sheet and the continuous ground metal, thus enabling fully interaction between analytes and hot spots. The corresponding field distributions in simulation corroborated our assumption and its sensitivity could be up to 1049 nm/RIU. Then, we fabricated the sample by e-beam lithography process for a seed layer and simply tilting the sample during deposition to obtain oblique flat sheets. When considering the stochastic nature of the deposited multiple oblique flat sheets, we modified the metallic upper resonator of the MPA from the single oblique-flat-sheet into randomly distributed oblique-wire-bundle (OWB) and in simulation, its sensitivity is boosted up to 3319 nm/RIU. In experiments, the measured sensitivity is 1329 nm/RIU under different concentrations of glucose solutions that is four times larger than the 330 nm/RIU of the planar MPA. The higher sensitivity was attributed to that the OWB MPA could provide hot spots within air not only between OWB and grounded metal but also among wires. Moreover, the OWB could also trap and concentrate the analytes locally.

## Introduction

Since metamaterials proposed by Pendry and Smith et al*.* emerged in 1999^1,2^, within these two decades, they became phenomenal success in different kinds of meta-devices and are widely applied in different fields, for example, invisibility cloaks for stealthy technology^3,4^, slow light for optical storage and computation^5–7^, metamaterial-based sensors for bio-sensing/chemical detection^8–10^. Among these meta-devices, metamaterial-based sensors are one of the most important developing devices due to the strong requirement for food safety detection^11–13^ and explosive detection^14^ and have attracted many researchers’ efforts on further improving their sensitivity for lower detection limit. When employed in the design of a sensor, metamaterials could provide concentrated electric fields adjacent to resonators, also known as hot spots; when analytes interact with these hot spots, the resonating frequency of metamaterials would red-shift, enabling the detection and analysis of the analytes. Based on this mechanism, to further enhance the sensitivity of metamaterial-based sensors, researchers tried to generate a very strong hot spot by different methods such as different resonator designs^8,15,16^, plasmonic nanoparticles^17,18^, gap plasmon^19–21^, and metamaterial perfect absorbers^8,10,15,22^. Plasmonic nanoparticles such as gold or silver nanoparticles obtained from chemical synthesis could provide ultra-strong electromagnetic field enhancement and large-area hot spots due to their small gaps among particles. Besides, the synthesis of nanoparticles is low cost and easy. On the other hand, the gap plasmons excited from the two adjacent metallic resonators also provide large field enhancement within the precisely controlled small gaps. Finally, metamaterial perfect absorbers consisted of a sandwiched metal-dielectric-metal structure concentrate the field at the dielectric spacer, thus also offering a strong hot spot. Nevertheless, the nanoparticles would suffer from the insufficiencies of random distribution of the particles, thus resulting in poor stability and repeatability and the gap plasmons also showed the disadvantages including small-area hot spots and less interaction between analytes and hot spots. It is the metamaterial perfect absorbers, one of the most promising candidates to achieve higher sensitivity.

Still, the hot spot would concentrate within the dielectric layers of the MPAs, thus hindering the full interaction between analytes and hot spots. The sensitivity of the planar MPAs are ranged around 225–1000 nm/RIU^8,23,24^; to solve this issue, some researchers proposed three dimensional metamaterials that provide a hot spot within air^22,25–31^; alternatively, researchers proposed a micro/nano-fluidic integrated MPA to locate the hots spots within the fluidic channel where analytes could fully interact with hot spots^32–35^. The authors also employed Fano-resonance induced by coupling between molecular absorption and resonance absorption from metamaterials to enhance its sensitivity and detection limit^35,36^. Moreover, Su et al*.* proposed a vertical-wall MPA based on novel e-beam lithography procedure, plasma-etching process and oblique deposition method^37^. The vertical-wall MPA is composed of gold/air/gold three-layered structure. The air channel could facilitate full interaction between hot spots and analytes, thus enhancing its sensitivity. Nonetheless, all the above-mentioned methods inevitably increased the fabrication difficulties which might lower the yield and stabilities of the devices. Also, the sensitivities are only a little bit better than or comparable to the planar MPAs, for example, 885/900 nm/RIU in Refs.^22^ and^27^ for 3D metamaterials, 3.5 THz/RIU at the frequency of 6.4 THz and 140 GHz/THz at ~ 0.8 THz in Refs.^32^ and^33^ for micro/nano fluidic MPAs. Therefore, in this work, we proposed a simple method to enhancing the sensitivity of a metamaterial-based sensor by tilting the substrate to a certain angle to conduct oblique deposition^38–42^ with a periodic seed layer fabricated by e-beam lithography process and results in an oblique-flat-sheet (OFS) metamaterial perfect absorber. The field would concentrate between the oblique sheet and ground, thus providing space for analytes to fully interact with the hot spots.

## Design and simulation

**Figure 1:**
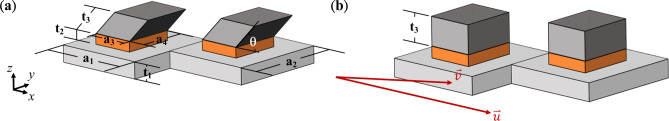
Schemes of (**a**) oblique-flat-sheet (OFS) and (**b**) planar metamaterial perfect absorbers. Here, a_1_ is 800 nm, a_2_ 850 nm, a_3_ 450 nm, a_4_ 700 nm, t_1_ 150 nm, t_2_ 120 nm, θ 40-degree and t_3_ 455 nm. Meanwhile, $\mathop{u}\limits^{\rightharpoonup} = 2\mathop{x}\limits^{\rightharpoonup}$ and $\mathop{v}\limits^{\rightharpoonup} = \mathop{x}\limits^{\rightharpoonup} + 0.5\mathop{y}\limits^{\rightharpoonup}$.

To achieve a unity absorption, the OFS MPA should satisfy the condition of destructive interference governed by $\tilde{r} = \tilde{r}_{12} - \frac{{\tilde{t}_{12} \tilde{t}_{21} e^{i2\beta } }}{{1 + \tilde{r}_{21} e^{i2\beta } }}$, whereas $\tilde{r}$ is the total reflection,$\tilde{r}_{12}$, $\tilde{r}_{21}$, $\tilde{t}_{12}$ and $\tilde{t}_{21}$ are reflection and transmission coefficients at the interface from the upper resonator to air and from air to the upper resonator, respectively. β is the propagation constant within the dielectric layer of the MPA^43,44^. Once $\tilde{r}$ is 0, we could achieve perfect absorption. To design the OFS MPA, all the dimensions should be chosen to meet the requirement for a guarantee of occurrence of the shadowing effect during oblique deposition and for an absorption band that is free of water absorption. Thus, we should first determine dimensions of the seed layer for the deposition of the oblique flat sheets. Here, to avoid residual metal deposition at the positions where no seed layers exist, unlike the conventional rectangular metamaterial array, we employed a parallelogram array with translation vectors of $\user2{\mathop{u}\limits^{\rightharpoonup} }$ and $\user2{\mathop{v}\limits^{\rightharpoonup} }$ equal to $2\mathop{x}\limits^{\rightharpoonup}$ and $\mathop{x}\limits^{\rightharpoonup} + { }0.5\mathop{y}\limits^{\rightharpoonup}$, respectively. Then, to confine all the deposited metal only on top of the seed layer, a coverage length for self-shadowing effect could be calculated by Eq. (1)^40^ shown below

1 $${\text{s}} = {\text{h}} \times \tan \left( \alpha \right)$$

where s denotes the coverage length of the self-shadowing effect, h the thickness of the seed layer and α the inclination angle of the substrate. Note that s should be always larger than the translational vector $\user2{\mathop{u}\limits^{\rightharpoonup} }$ along the x-direction of the MPA to guarantee no metal would grow outside the seed layer. Next, the inclination angle α would influence the maximum achievable angle β, the angle between the oblique flat sheet and grounded metal’s normal; such angles vary with different choices of metal. In this work, as shown in Fig. 1a, the periodicities of the MPA along the x- and y-direction are 800 and 850 nm, respectively. The thickness of grounded metal is 150 nm and the length, width and thickness of the seed layer are 455, 700 and 120 nm, respectively. The maximum angle β is 50-degree obtained from the experiments while α is 86-degree; thus, in simulation, the angle θ between the oblique flat sheet and the substrate (i.e., θ = 90 – β) is set to 40 degree. The seed layer is composed of MgF_2_ with permittivity of 1.8868 and loss tangent of 0.01. It is worth mentioning that the calculated coverage range for the parameter is 1716 nm, which is larger than 1600 nm and guarantees limited metal deposition only on top of the seed layer. Besides, the side-length of the oblique-flat-sheet is 455 nm. Finally, to examine the effect of sensitivity enhancement from our proposed method, we also simulate a planar MPA with the same parameters as the OFS MPA in order to make a fair comparison as shown in Fig. 1b. Note that we employed CST Microwave Studio with an algorithm of finite integration method and in simulation, the boundary conditions we employed are open and unit cell parallel and perpendicular to the propagation direction, respectively.

**Figure 2:**
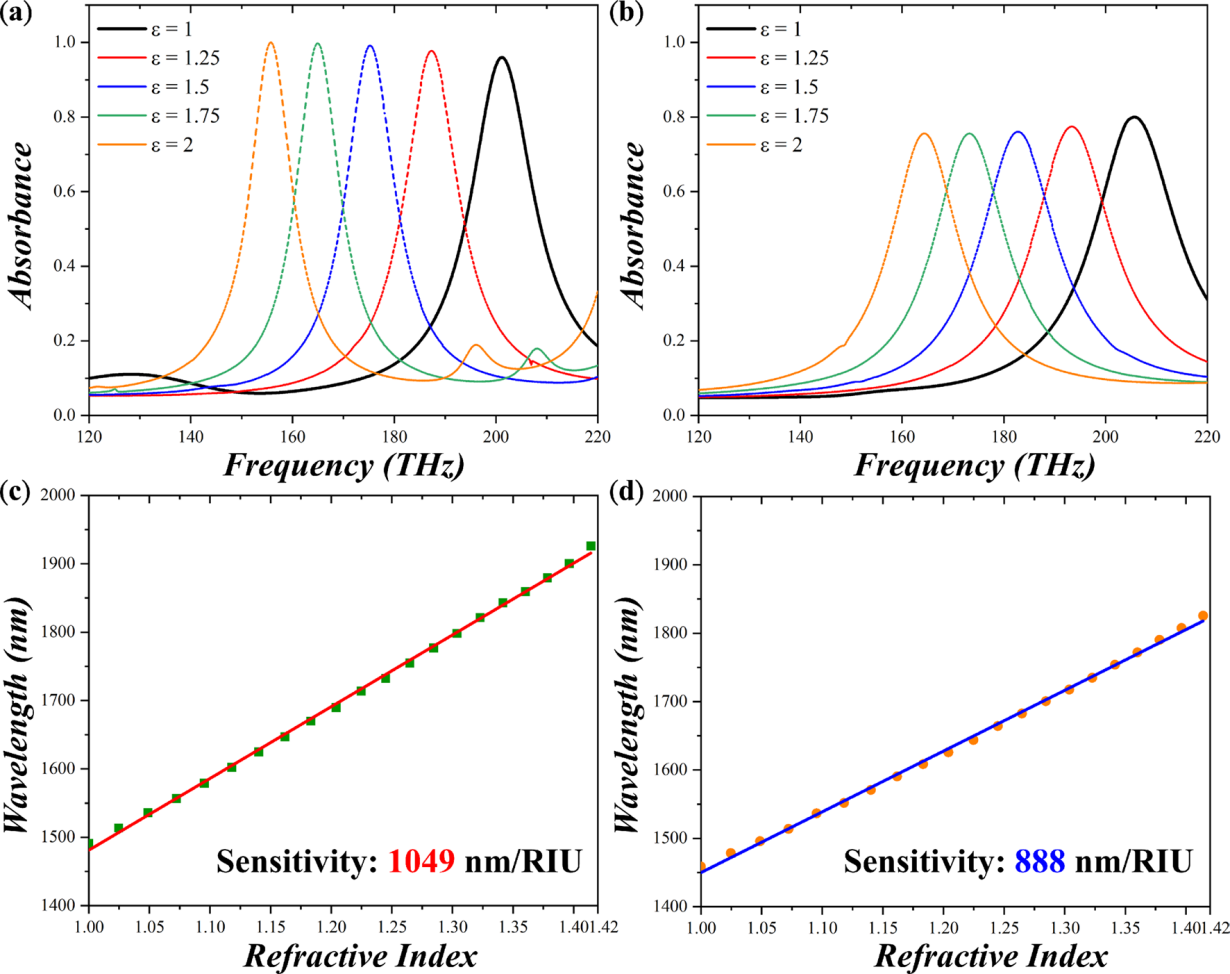
Absorbance of (**a**) the OFS and (**b**) the planar MPAs under environments with different refractive indices from 1 to 2. Resonant wavelengths with respect to the refractive index for (**c**) the OFS and (**d**) the planar MPAs. The corresponding sensitivity is 1049 and 888 nm/RIU, respectively.

**Figure 3:**
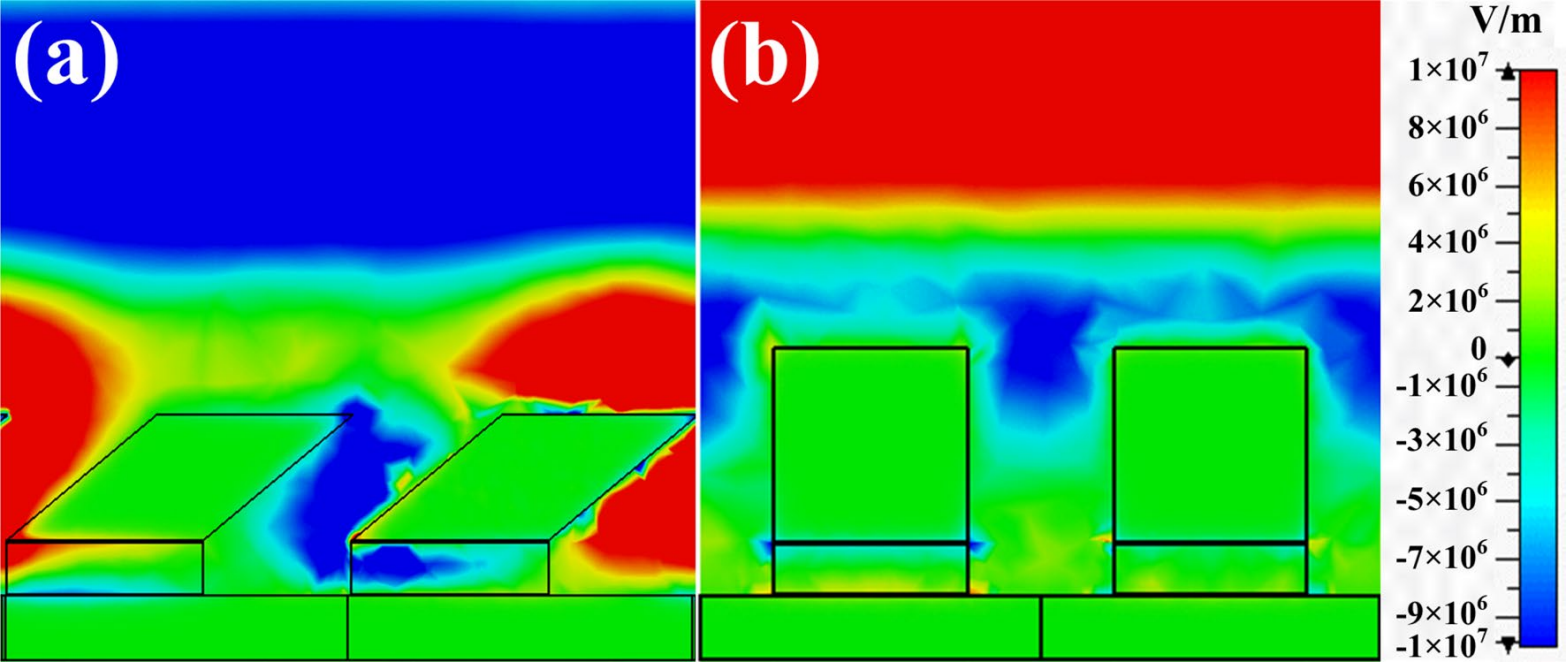
Field distributions of (**a**) the OFS MPA at 201.69 THz and (**b**) the planar MPA at 205.43 THz. The OFS MPA revealed stronger electric field and larger area hot spots compared to the ones of planar MPA.

The absorbance spectra of the OFS MPA and planar MPA are depicted in Fig. 2a, b, respectively; there appear significant absorbance peaks at the frequencies of 201.69 and 205.43 THz with a magnitude of 94.57% and 78.16%, respectively. Then, we applied analytes on the MPAs with the refractive index changing from 1 to 2 and the absorbance peaks of the both MPAs are red-shifted. The sensitivity (${\text{S}} = \Delta \lambda /\Delta n$ where $\Delta \lambda$ is resonance wavelength change while $\Delta n$ is refractive index change) of the OFS MPA is 1049 nm/RIU that is higher than 888 nm/RIU of the planar MPA as depicted in Fig. 2c, d. It is 1.18 folded enhancement compared to the planar MPA. Taking a step further, we calculated the figure of merit (${\text{FoM}} = { }\frac{Sensitivity}{{Bandwidth}}$) for the both MPAs. The FoM for the OFS and planar MPAs are 9.52 and 7.90. The higher sensitivity and larger FoM reveal the superior sensing behavior of our proposed OFS MPA compared to the planar one. To further dig into the hidden mechanism, we monitored the electric field distribution at the frequency of the absorbance peaks. From Fig. 3a, b, we clearly observe that for the OFS MPA, the hot spots indeed located within the air regime between the oblique flat sheet and grounded metal, thus fully interacting with analytes and resulting in better sensitivity. In contrast, the hot spots of the planar MPA concentrated on the dielectric layers, providing limited interaction with analytes and thus smaller sensitivity.

## Fabrication and measurement

**Figure 4:**
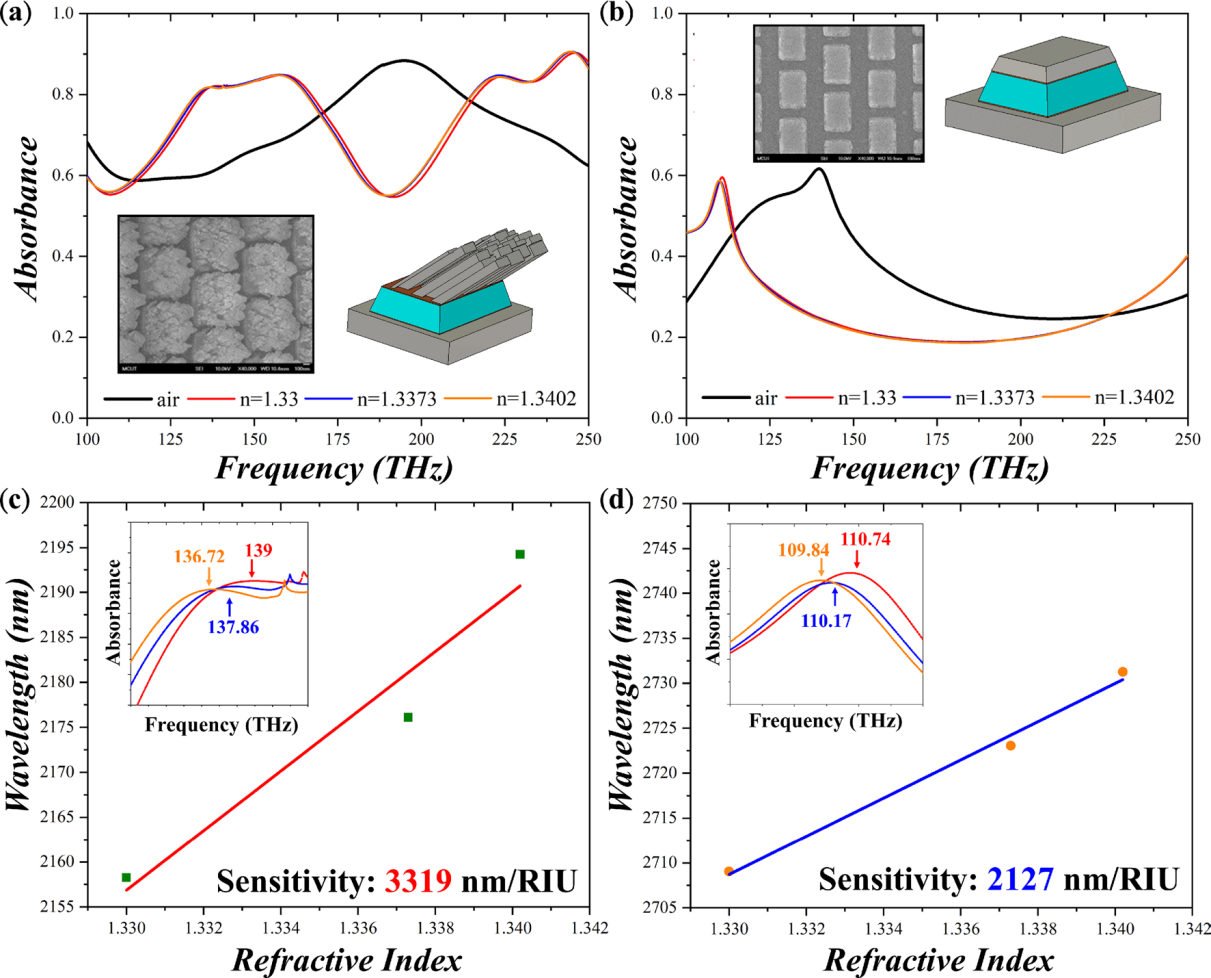
Absorbance of (**a**) the oblique-wire-bundle (OWB) and (**b**) the planar MPAs under ambient environments and different concentrations of glucose solutions with refractive index from 1.33 to 1.3402. Insets depict SEM images of the as-fabricated samples and their modified morphologies in simulation. Resonant wavelengths with respect to the refractive index for (**c**) the OWB and (**d**) the planar MPAs. The corresponding sensitivity is 3319 and 2127 nm/RIU, respectively. Insets show enlarged spectra near resonant frequencies.

**Figure 5:**
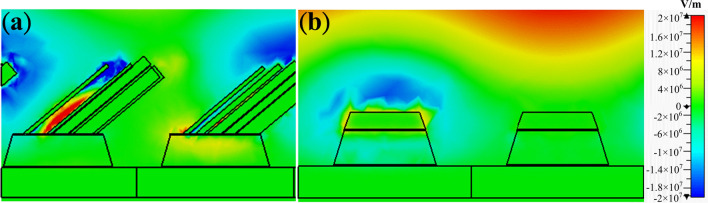
Field distributions of (**a**) the OWB MPA at 195.18 THz and (**b**) the planar MPA at 138.96 THz. The OWB MPA revealed stronger electric field and larger area hot spots compared to the ones of planar MPA. Note that the gaps among nanowires also provided hot spots, thus further enhancing the sensitivity of the MPA.

To fabricate the sample, we conducted electron beam vapor deposition and e-beam lithography for deposition and patterning, respectively to make a periodic array to mimic the unit cell boundary condition in simulation (see “Methods” section for details). Then, the sample was put on a holder that was tilted an angle of 86-degree to the horizon for oblique deposition. To compensate potential manual error when putting the sample on the holder, we increased the thickness of the seed layer up to 166 nm (160 nm MgF_2_ and 6 nm Ti), thus guaranteeing a coverage length of 2374 nm. SEM images of the as-fabricated sample are illustrated in the inset of Fig. 4. Compared to the simulation, the as-fabricated sample revealed a stochastic distribution of nano-wire bundles on top of the seed layers. Also, the dimensions deviate to the simulated one because of the inevitable fabrication errors from the lithography process. Furthermore, due to undercut of the resist, the seed layer, instead of a cuboid, became a frustum of a rectangular pyramid with a tilted angle of 16-degree of the side length. To reflect the morphology of the fabricated sample, in simulation, the periodicities along x- and y-direction became 795 nm and 855 nm; the length and width of the seed layer were 550 and 740 nm. The side length of the oblique wire bundles is around 290–350 nm with a random distribution. On the other hand, the planar MPA also shows some dimensional deviation including the periodicities of 810 and 855 nm along x- and y-direction, the length of 480 and width of 700 for the seed layer. Here, the seed layer also became a frustum of a rectangular pyramid with a tilted angle of 16-degree of the side length. In addition, due to the fabrication difficulty, we reduced the thickness of the upper metal down to 80 nm. Figure 4a, b shows the simulated absorbance for the OWB and planar MPA, respectively (see Supplementary Materials for polarization and incident angle dependence of the OWB MPA). There appears an absorbance peak at 195.18 THz for the OWB MPA and at 138.96 THz for the planar MPA. Here, we could observe that the OWB MPA revealed broader absorption bandwidth compared to the planar MPA. Such broad absorption could be attributed to two factors. One is that since OWB MPA possessed many wires instead of a single flat sheet and each wire could contribute absorption at different but similar frequencies, thus the OWB MPA showed broad absorption bandwidth. The other is that the coupling among wires might also contribute to the broadening of absorption bands^42^. Besides, although the OWB MPA is asymmetric, still the cross-polarization reflectance is small (~ 4%) compared to co-polarization reflectance. Here, we further simulated the frequency change with the addition of deionized water and glucose solutions. The refractive indices of the deionized water and 3% and 5% glucose solutions are 1.33, 1.3373 and 1.3402, respectively. The corresponding sensitivity is 3319 nm/RIU and 2127 nm/RIU for the OWB and planar MPAs as illustrated in Fig. 4c, d. The sensitivity of the OWB MPA is 1.56 times the value of the planar MPA, evidencing the usefulness of the proposed method for sensitivity enhancement. Note that since the lower resonance frequency guaranteed better sensitivity, thus the enhancement of sensitivity could be larger from the OWB MPA if the resonating frequencies of the two are the same^10^. Again, the corresponding field distributions of the OWB and planar MPAs are recorded and plotted in Fig. 5a, b at the frequencies of 195.18 and 138.96 THz. Overall, the field of the planar MPA is much weaker compared to the one of the OWB MPA. Also, the OWB MPA could provide the larger area of the hot spots. Further, the fields concentrate not only between the oblique wires and grounded metal plane but also among nanowires, providing much more hot spots than the OFS MPA.

**Figure 6:**
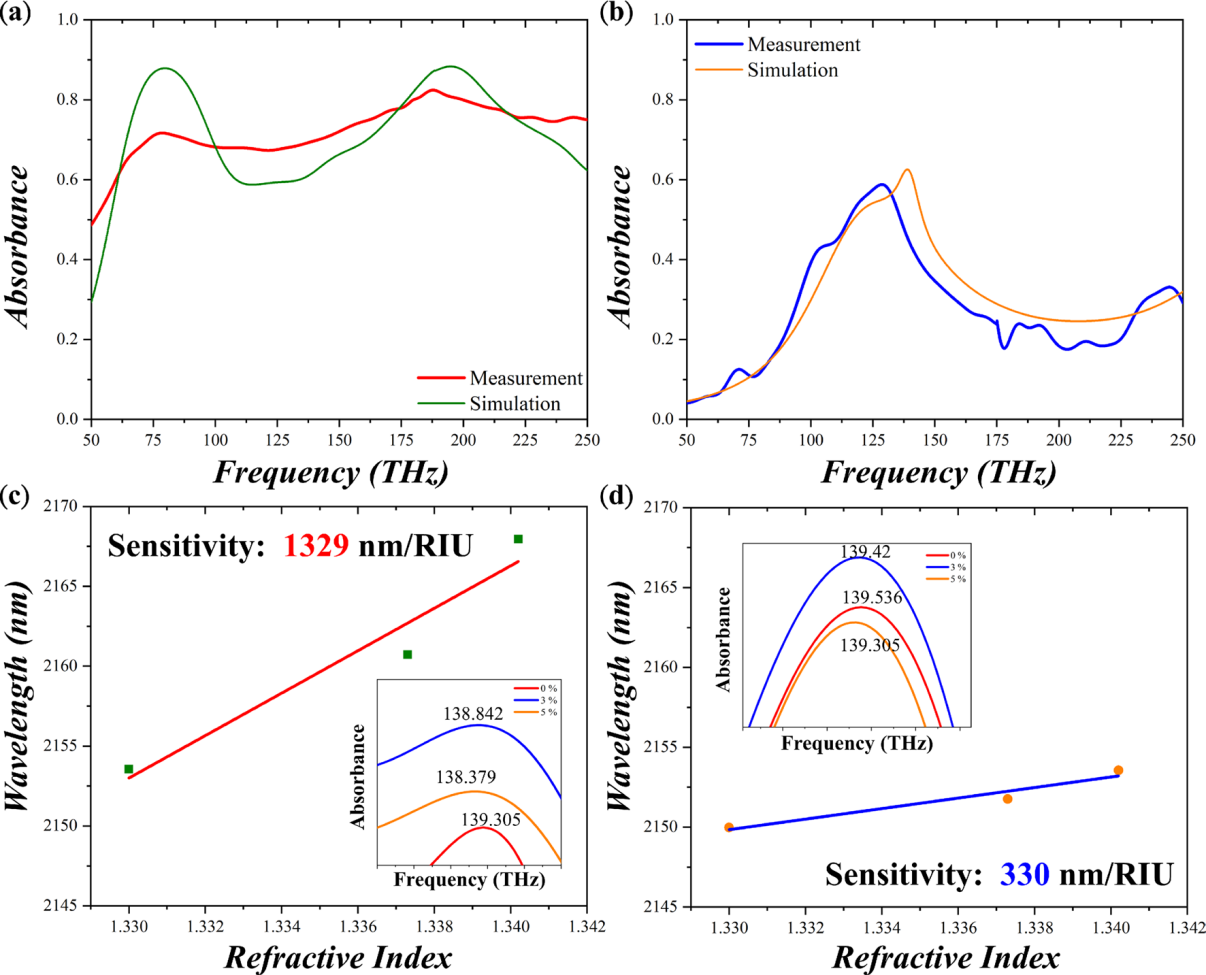
Measured absorbance spectrum of (**a**) the OWB MPA and (**b**) the planar MPA. Measured results agree the simulated ones well. The resonant wavelength with respect to refractive index for (**c**) the OWB MPA and (**d**) the planar MPA with the sensitivity of 1329 nm/RIU and 330 nm/RIU, respectively. Insets depict the enlarged spectra near the resonance frequency.

To measure absorbance of the fabricated MPAs, we employed μ-Fourier-transformed infrared spectroscopy (Bruker V70 equipped with a microscope), the light spot was focused onto the MPA area equal to 100 × 100 μm^2^. The measured absorbance spectra are illustrated in Fig. 6a, b for the OWB and planar MPAs. The simulated spectra are also included. The first two absorbance peaks of the OWB MPA correspond to the simulated results well and so does the absorbance peak of the planar MPA. Still, some deviations such as smaller absorbance of the two peaks and larger absorbance for the gap between the two peaks for the OWB MPA and minor absorbance peaks for the planar MPA. Such deviation could be attributed to the different permittivity dispersion and dimensional variation in simulation and in experiments. Next, we used deionized water, and 3% and 5% glucose solutions as the analytes to test the sensitivity of the two MPAs. By recording its resonance wavelength change with respect to the refractive index change under different concentrations of glucose solutions, we can retrieve the sensitivity in experiments. Unfortunately, due to strong water absorption from water, only frequency shifts around 140 THz could be observed. The corresponding sensitivity of the OWB MPA is 1329 nm/RIU which is 4 times larger than 330 nm/ RIU of the planar MPA as shown in Fig. 6c, d, respectively. Such difference could be related to the location and the area of the hot spots. It is worth mentioning that the smaller sensitivity in experiments than in simulation might originate from the fabrication error and high losses from metal by oblique deposition method. Moreover, when scrutinizing the simulated and measured sensitivity, the measured sensitivity of the planar MPA degraded much severely compared to the one of the OWB MPA. Such behavior might originate from that in experiments, due to stochastically distributed oblique wires, the structure could provide a trap for the analytes and concentrate the analytes locally. This locally higher concentration would induce larger frequency shift and so does the sensitivity. On the contrary, the planar MPA cannot trap analytes, thus showing a significant degradation of the sensitivity. Finally, we tabulated the sensitivity of different metamaterial perfect absorbers in Table 1 for comparison. In this summary, our proposed OWB MPA possessed higher sensitivity not only in simulation but also in experiments.

**Table 1:** Comparison of the sensitivity of metamaterial perfect absorbers.

Work	Upper resonator	Operating frequency (THz)	Sensitivity (nm/RIU)
Our work	Oblique-wire-bundle	139	3319 1329*
Ref.^45^	Planar ring/disk	150	225
Ref.^23^	Split-rings	193	500
Ref.^24^	C-shaped spit-ring with a thin wire	390	1000
Ref.^46^	Suspended microracetrack	150	337.5
Ref.^47^	Triangle cavity coupled with ellipse-ring	350	860
Ref.^48^	Planar square array	236	840
Ref.^29^	3D split ring	280	300
Ref.^31^	3D Gold disk with aluminum pillars	556	683.5*

Note that * indicated experimentally measured sensitivity.

## Conclusions

With the abovementioned simulation and measurement results, we designed, and simulated the oblique-flat-sheet metamaterial perfect absorber. The OFS MPA shows sensitivity of 1049 nm/RIU. Then, we conducted e-beam deposition and lithography procedure to fabricate the sample. To take the dimensional change of the as-fabricated sample into consideration, in simulation, we amended the structure into a stochastically-distributed oblique-wire-bundle MPA based on the SEM image and such modifications resulted in sensitivity up to 3319 nm/RIU, almost 3.2 folded enhancement. In addition, the sample was characterized by μ-FTIR and revealed a good agreement between the measured and simulated spectra. Finally, to test the experimental sensitivity, deionized water, and 3% and 5% glucose solutions were applied onto the sample. The sensitivity is 1329 nm/RIU which is 4 times larger than 330 nm/ RIU of the planar MPA. To conclude, we provide, in this work, a simple method, by just tilting the sample an angle with respect to the horizon, to boosting the sensitivity of the MPA-based sensors. We believe this work would pave the route toward future advanced bio-sensing and bio-imaging.

## Method

### E-beam lithography and deposition procedure

To construct an OWB MPA, a silicon substrate was cleaned on which 150-nm-thick aluminum was deposited. Then, A5 PMMA resist was spin-coated with a rotation speed of 1000 rpm for 10 s and then a rotation speed of 4000 rpm for 60 s. Prebake the sample up to 180 °C for 3 min. Next, the sample was exposed to electrons with a dosage of 200 μC/cm^2^ and then immersed in the developed solution (MIBK:IPA = 1:3) for 50 s. In the following, 6-nm-thick titanium, 160-nm-thick MgF_2_ and 6-nm-thick titanium were deposited in sequence onto the sample. Furthermore, lift-off process was carried out by immersing the sample into the acetone solution for a day and then the beaker with the sample was put inside an ultrasonic vibrator for 2 min with a frequency of 80 kHz and power efficiency of 40%. The sample was then attached to a holder that tilted 86-degree with respect to the horizon in the e-beam evaporator with an operating pressure of 6 × 10^–6^ torr. The reading from the quartz oscillator was 500 nm for aluminum deposition. Similar fabrication procedure was conducted for the planar MPA except that during deposition, the upper resonator was deposited continuously after the deposition of the seed layer.

## Supplementary Information

Supplementary Information.
