# Supplementary material for: Creating hot spots within air for better sensitivity through design of oblique-wire-bundle metamaterial perfect absorbers
Source: Sci Rep. 2022 Mar 3;12:3557. doi: 10.1038/s41598-022-07338-6 (PMC8894341; doi:10.1038/s41598-022-07338-6)
Supplement: Supplementary file 1 — Supplementary Information. [file 41598_2022_7338_MOESM1_ESM.docx]

**Creating Hot Spots Within Air for Better Sensitivity through Design of Oblique-wire-bundle Metamaterial Perfect Absorbers**

**Xin-Xian Wu^1^, Cheng-Yu Lu^1^ and Tsung-Yu Huang^*, 1^**

^1^Department of Materials Engineering, Ming Chi University of Technology, New Taipei City 243303, Taiwan

^*^huang.tsungyu@mail.mcut.edu.tw

- **Polarization dependence of the oblique-wire-bundle metamaterial perfect absorber**

Our proposed OWB MPA possessed two absorption bands for each polarization. At y-polarization, the absorption bands are located at 79.70 and 194.94 THz with absorbance of 87.86% and 88.35%, respectively. On the other hand, at x-polarization, the absorption bands are located at 82.43 and 233.34 THz with absorbance of 62.78% and 95.26%, respectively. The polarization dependence of the OWB MPA is also plotted as shown in Fig. S1. Note that the resonance frequency of the first absorption band is similar but with gradually decreasing absorbance under different polarization; in contrast, the resonance frequency of the second absorption band shifted to higher frequency with gradually decreasing absorbance till 45-degree and then increasing again till 90-degree.

**
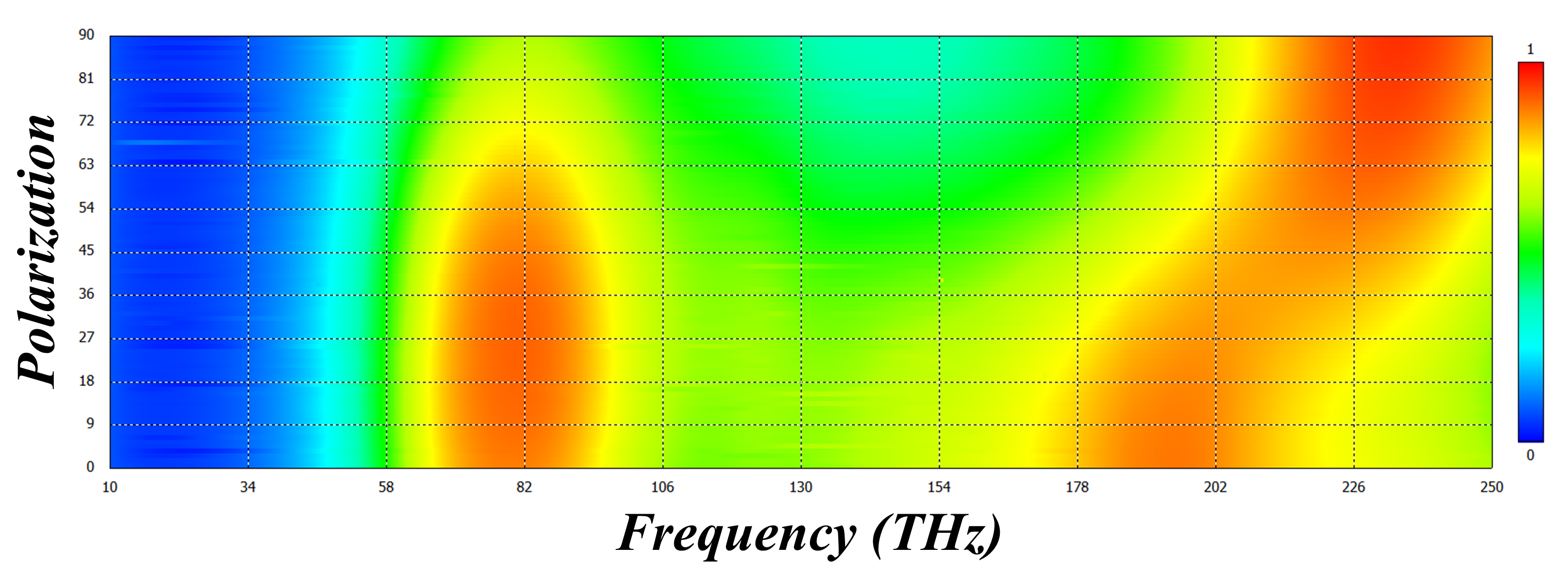
**

**Figure S1.** Simulated absorbance colormap with respect to the polarization angle (from y- to x-polarization) and frequency.

- **Incident angle dependence of the oblique-wire-bundle metamaterial perfect absorber**

To consider the incident angle dependence of our proposed OWB MPA, the absorbance colormaps with respect to the incident angle and frequency are recorded in simulation for both TM and TE modes. For the TM mode, the absorbance peak shifted to the lower frequency while the higher-order reflectance appeared. On the other hand, for the TE mode, the resonance frequency of the two absorption bands are almost intact. Also, the absorbance of the first absorption band remained above 80% before 75-degree; yet, the absorbance of the second absorption band gradually decreased. Finally, higher-order reflection could be also observed at higher frequency and high incident angles.

*
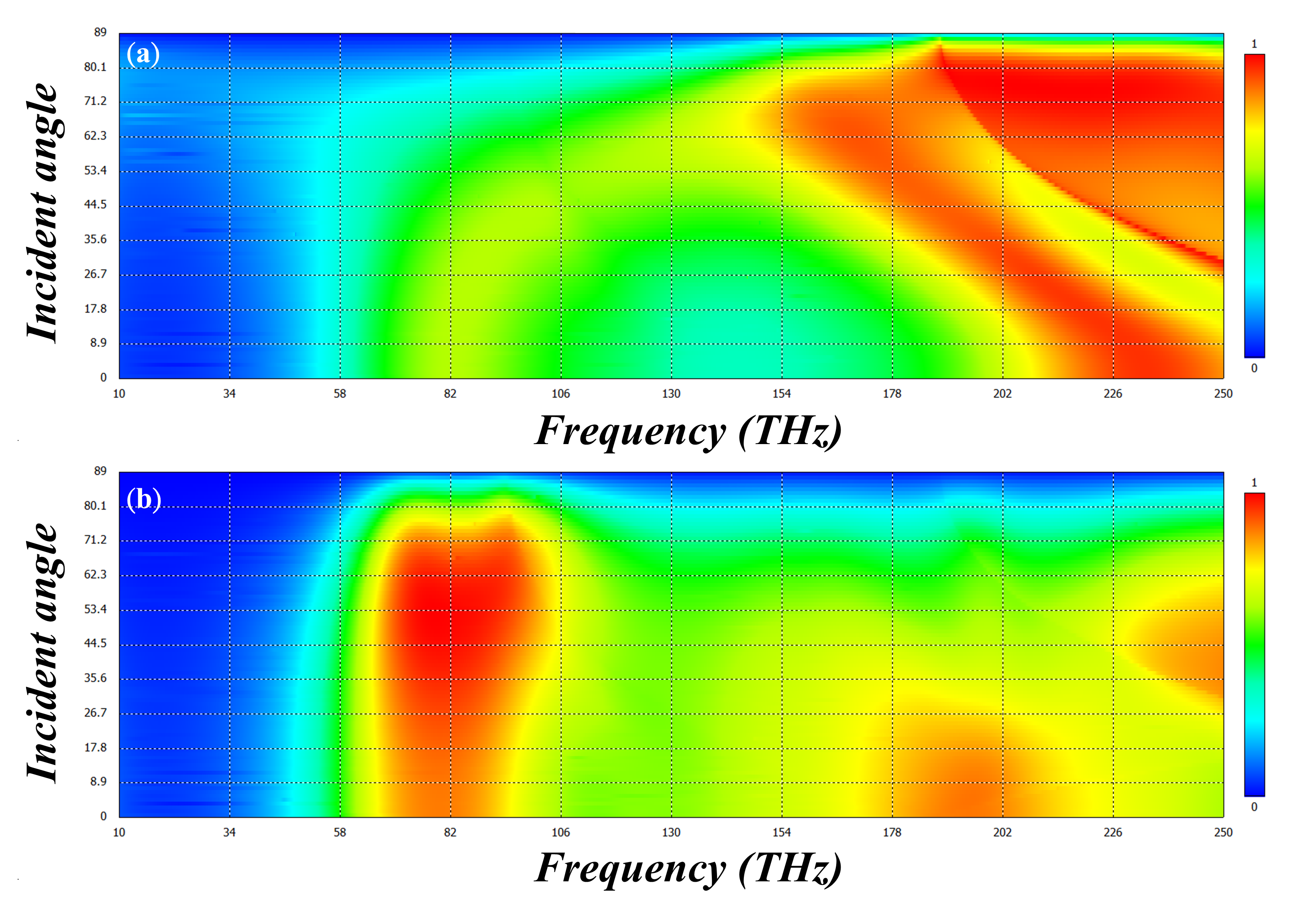
*

**Figure S2.** Simulated absorbance colormaps with respect to incident angles and frequency for (a) TM and (b) TE modes, respectively.
